# Density Functional Theory Studies on the Synthesis of Poly(α-Amino Acid)s Via the Amine-Mediated Ring Opening Polymerizations of *N*-Carboxyanhydrides and *N*-Thiocarboxyanhydrides

**DOI:** 10.3389/fchem.2021.645949

**Published:** 2021-03-29

**Authors:** Tianwen Bai, Botuo Zheng, Jun Ling

**Affiliations:** MOE Key Laboratory of Macromolecular Synthesis and Functionalization, Department of Polymer Science and Engineering, Zhejiang University, Hangzhou, China

**Keywords:** polypeptide, polypeptoid, quantum chemical calculation, mechanism and kinetic, polymer synthesis

## Abstract

To synthesize well-defined poly (α-amino acid)s (PAAs), ring opening polymerizations (ROP) of cyclic monomers of α-amino acid *N*-carboxyanhydrides (NCAs) and *N*-thiocarboxyanhydrides (NTAs) are most widely used. In this mini-review, we summarize the mechanism details of the monomer preparation and ROP. The present study used density functional theory calculations to reveal the mechanisms together with experimental phenomena in the past decades. Detailed discussion includes normal amine mechanism and the selectivity of the initiators bearing various nucleophilic groups.

## Introduction

Poly(α-amino acid)s (PAAs) are promising biomaterials that have been employed in drug delivery, gene engineering, and self-assembly fields in recent years (Deming, 2016; Gangloff et al., 2016; Tao et al., 2018a; Birke et al., 2018). The synthetic process toward PAA includes ring opening polymerization of a cyclic monomer such as α-amino acid *N*-carboxyanhydrides (NCAs) and *N*-thiocarboxyanhydrides (NTAs), solid-phase synthesis, and biological methods (Mazo et al., 2020). NCAs were first prepared by Leuchs (Leuchs, 1906) in 1906, and NTAs were first reported by Aubert and Knott (1950). The ring opening polymerization (ROP) of cyclic monomers is still the most promising synthetic route toward PAAs. Due to the existence of multiple active sites located on NCA or in the NTA ring, including carbonyl groups at 2- and 5-positions for nucleophilic attack and acidic protons of 3-NH and 4-CH groups, various initiators have been carried out for polymerization including amines (Kricheldorf, 1987), trimethylsilyl (TMS) containing compounds, (Lu and Cheng, 2007, 2008; Yuan et al., 2016; Baumgartner et al., 2017; Yuan et al., 2018), salts (Szwarc, 1965; Wu et al., 2018), (activated) alcohols (Zhao et al., 2019; 2020), *N*-heterocyclic carbene (Guo and Zhang, 2009; Falivene et al., 2016; Falivene and Cavallo, 2017), transition metal catalysts (Deming, 1997), rare earth complexes (Ling et al., 2012; Peng et al., 2012; Tao et al., 2014), and so on.

**SCHEME 1:**
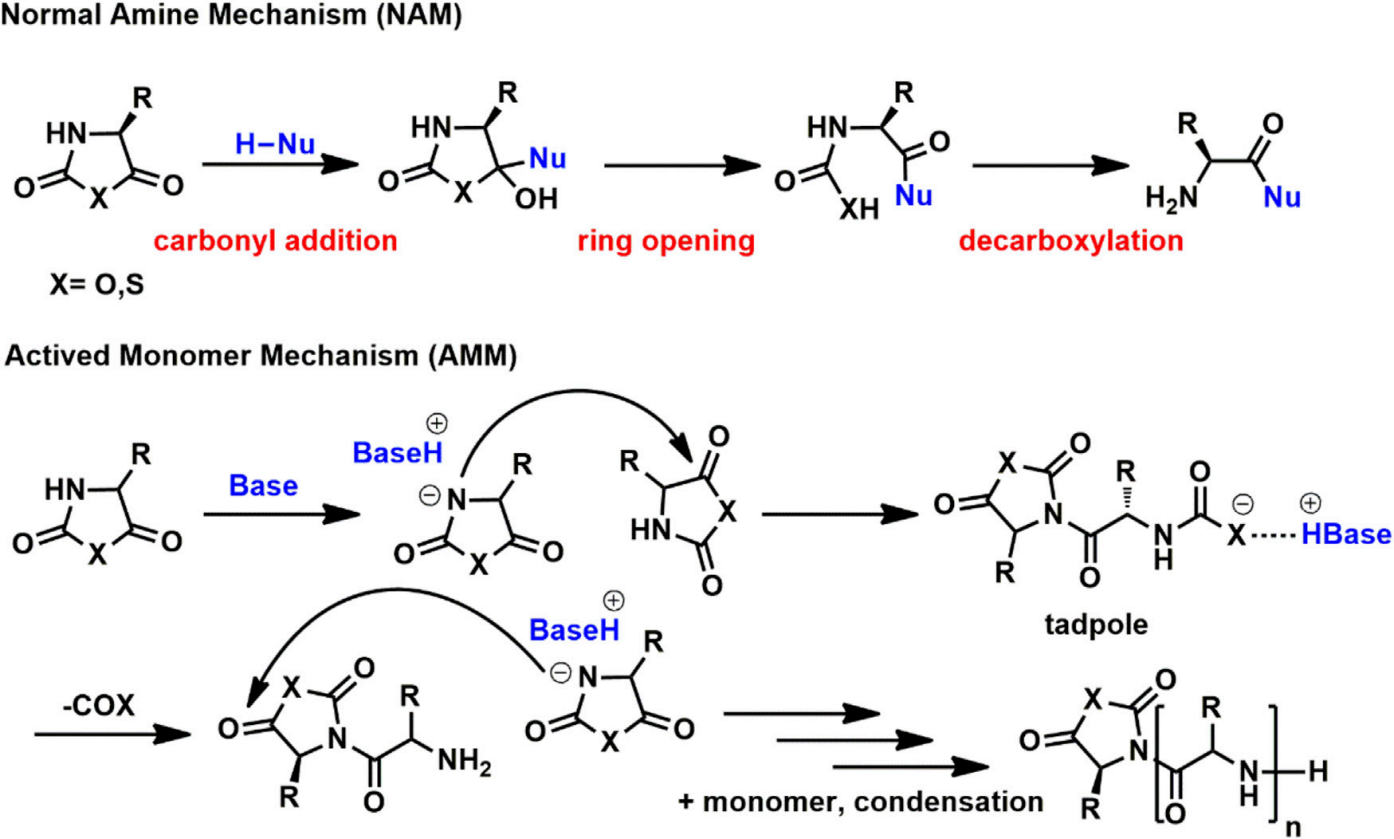
NAM and AMM mechanisms for the ring opening polymerization of cyclic monomers toward poly(α-amino acid)s.

The diversity of initiators leads to the complexity of ROP mechanisms, including normal amine mechanism (NAM), active monomer mechanism (AMM), carbene mechanism and so forth (Kricheldorf, 2006). NAM is considered responsible for living/controlled ROP, which follows chain propagation mechanism. Initiators in NAM usually bear at least one labile proton, as shown in Scheme 1. In mechanism investigation, NAM consists of three major steps, i.e., carbonyl addition, ring opening and decarboxylation. The rate-determining step of NAM is still under debate which is carbonyl addition or decarboxylation depending on monomers, initiators, and polymerization conditions. Differing from NAM, strong basic initiators prefer AMM, which is capable of ionizing cyclic monomers *via* deprotonation on 3-NH. As a result, AMM is only suitable for NCA and NTA but not *N-*substituted glycine NCA (NNCA) and the corresponding NTA (NNTA) monomers since they do not contain a proton on nitrogen atom.

The experimental phenomena of AMM of NCA and NTA are similar to those of the general AMM of cyclic monomers, for instance, fast propagation rate, high monomer conversion in a short time, high molecular weight (MW), and broad dispersity of product, *etc*. Fast polymerization rate also makes AMM a practical method to prepare high-MW PAAs regardless of limited solubility. The details of AMM are not well-understood yet and lack information on reaction and overall kinetics analyses. AMM and NAM are sometimes found mixed, such as metal complex catalytic systems or initiation by mixtures of primary and tertiary amines.

For amine initiators, NAM and AMM have a boundary based on nucleophilicity and basicity. Primary amines follow NAM and tertiary amines follow AMM, but secondary amines depend on substituted groups. For linear substituted secondary amines such as propagation end in NNCA polymerization, good controllability and pseudo first kinetics are confirmed, but for some α-branched substituted secondary amines such as diisopropylamine obeys AMM. The nucleophilicity and basicity of amines are also influenced by interaction with other groups, especially in α,ω-telechelic initiators such as α,ω-alcohol/thiol amine (Tao et al., 2017; Tao et al., 2018b; Zhao et al., 2019; 2020).

Limited by our experimental tools, the molecular scale understanding of the mechanism is still lacking. Quantum chemistry tools such as density functional theory (DFT) can provide understanding at the molecular level, which could explain the essentials of NAM and AMM. In the present work, DFT investigation for monomer synthesis and NAM details are reviewed with some comments on unsolved mechanism problems.

### Synthesis of NCA and NTA Monomers

**FIGURE 1:**
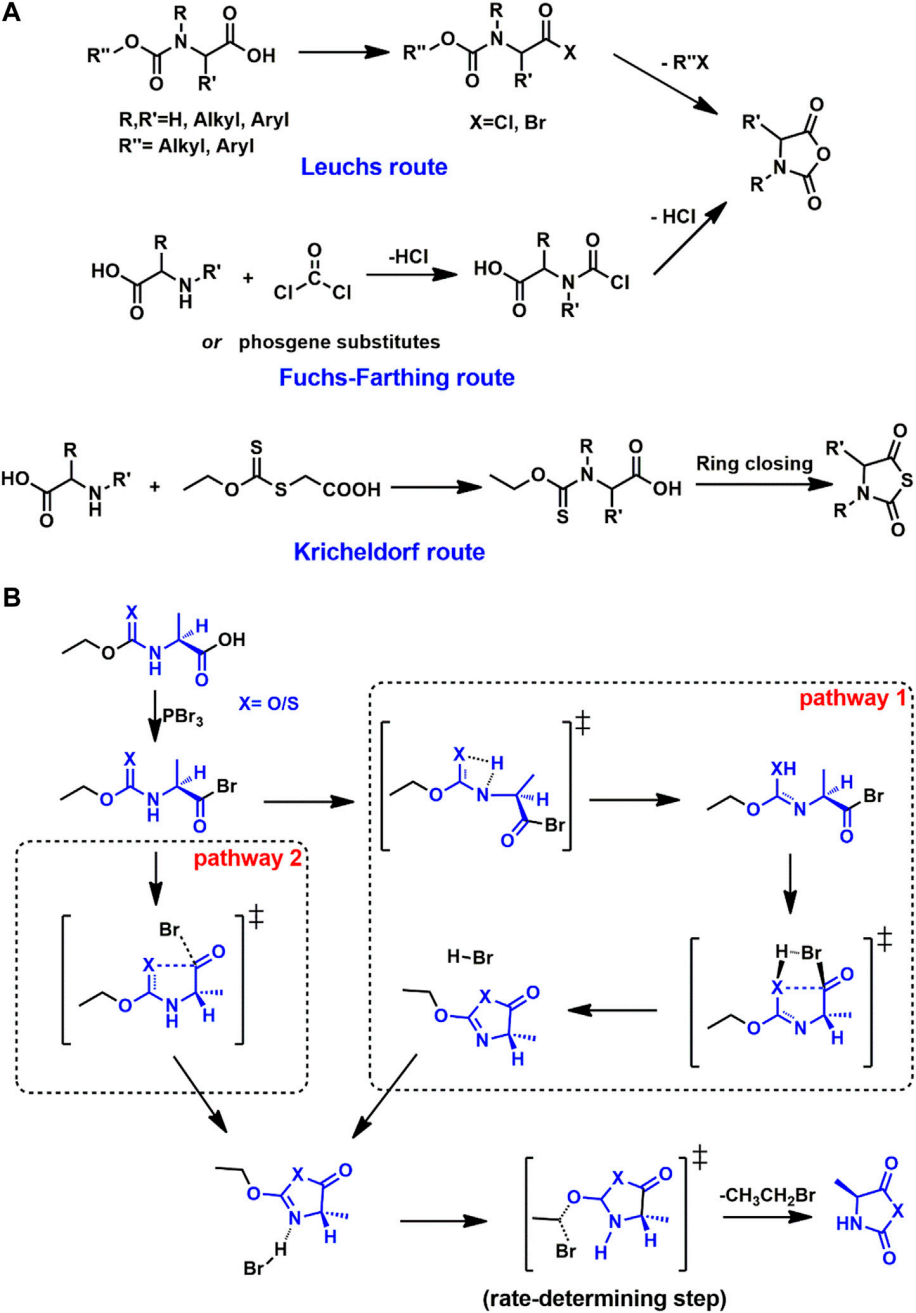
**(A)** Syntheses of NCA monomers *via* Leuchs and Fuchs-Farthing routes and NTA monomers *via* Kricheldorf route. **(B)** Two possible ring-closing pathways toward Ala-NCA and Ala-NTA following Leuchs route or Kricheldorf.

The preparation of cyclic monomers is the first step toward PAAs. Hermann Leuchs synthesized NCAs from *N*-ethoxycarbonyl and *N*-methoxycarbonyl chloride *via* cyclization upon heating in vacuo. Such cyclization of *N*-alkoxycarbonyl halogenides toward NCAs is hence known as the *Leuchs method* (Leuchs, 1906; Leuchs and Manasse, 1907; Leuchs and Geiger, 1908), shown in Figure 1A. However, the Leuchs method cannot produce *N*-dinitrophenyl or *N*-acyl NCAs (Kricheldorf, 1987). The simplest and most widely-used method for NCA synthesis is a direct reaction between corresponding free amino acids with phosgene or its derivatives known as the *Fuchs-Farthing* route (Coleman and Farthing, 1950; Farthing and Reynolds, 1950). Benefiting from stability, NTAs can be carried out in an aqueous solution. Similar to the Leuchs route, the cyclization of *N*-ethoxythiocarbonyl amino acids with PBr_3_ or other ring-closing reagent was first carried out by Kricheldorf and Bösinger (Kricheldorf and Bösinger, 1976).

The critical compound in both the Leuchs and Kricheldorf routes is the *N*-alkyl(thio)carbonyl halogenides, which is too active to be caught in experimental work. After the ring closing process, some possible five-membered ring structures are proposed (Hirschmann et al., 1971; Vinick and Jung, 1982; Montalbetti and Falque, 2005). To investigate the synthetic details from the *N*-alkyl(thio)carbonyl halogenides to corresponding cyclic monomers, DFT calculations have employed model compound derived from alanine- (Ala-) and sarcosine- (Sar-) NCA or NTA (Bai et al., 2020). Two possible pathways are put forward as shown in Figure 1B.

For both NCA and NTA monomers, pathway 2 is preferred according to the comparison of the highest Gibbs free energy barriers in the whole process. In pathway 2, after the ring closing transition state (TS), the equivalent bromide anion is released. The bromide anion would further attack the methylene moiety neighboring oxygen with an S_N_2 substitution, which produces the final cyclic monomer *via* the corresponding TS. In the case of Ala-NCA, the rate-determining step is the S_N_2 step with a Gibbs free energy barrier of 22.0 kcal/mol under M06-2X/6-311++G(d,p) in the solvation model of dichloromethane. For Ala-NTA, the energy barrier of S_N_2 is 24.6 kcal/mol, which is a little higher than that of Ala-NCA.

A systemic study was carried out involving the effect of halides and *N*-substituents. As the starting reagents in the ring closing step, PBr_3_ is proposed to be a more active reagent than PCl_3_. DFT calculations confirm this based on the TSs along the pathways, with a benchmark analysis based on M06-2X, BHandH, and B3PW91/6-311++G(d,p). In TS of S_N_2 reaction, bromide is observed with longer interaction distance and more negative charge than chloride. For NNCA and NNTA, pathway 1 is forbidden due to the absence of hydrogen on nitrogen. Similar reaction pathways are found in the syntheses of alanine-NTA (Ala-NTA) and sarcosine-NTA (Sar-NTA), where Sar-NTA is confirmed with a lower energy barrier (18.5 kcal/mol) in the S_N_2 step compared with Ala-NTA (24.6 kcal/mol).

Racemization of NTA is of great importance due to the related chiral properties and secondary structures. The strong protonic acid is responsible for the racemization with S_N_2 TS, and the bulky electron cloud near α-C will suppress the racemization according to DFT calculations. For instance, leucine-NTA and isoleucine-NTA are harder to undergo racemization than Ala-NTA. The energy barrier (28.6 kcal/mol) of racemization is also noteworthy as it is higher than that of the synthesis of Ala-NTA (24.6 kcal/mol), which indicates that the racemization can be suppressed if shortening the reaction time.

### Progress in Amine-Initiated NAM

In the last century, the search for the rate-determining step of NAM has become of great importance to research, since knowing it will enable chemists how to improve polymerization rate and controllability (Kricheldorf, 2006). Experimental results indicate that the rate-determining step can be one of carbonyl addition and decarboxylation, but still lacks sufficient evidence to conclude and may depend on initiators, monomers, and even polymerization conditions (Kricheldorf, 1987). For example, the polymerization is faster under an inert atmosphere, i.e., inert gas purged or in a vacuum, compared with that in sealed rubes for some NCAs (Habraken et al., 2011; Zou et al., 2013; Fan et al., 2014). Some NCAs are not (Habraken et al., 2011). It is also suspected that the dissolved CO_2_ decreases the reaction rate (Tao et al., 2014; Cao et al., 2017; Siefker et al., 2018). In some copolymerizations, it is the monomer species that determine the reaction rate (Shalitin and Katchalski, 1960). To provide a molecular scale understanding, DFT calculations do help. The first calculation work upon NAM was carried out by Ling and Huang (2010), taking _L_-Ala-NCA initiated by ethylamine as a model reaction. The NAM is divided into three main steps, i.e., carbonyl addition, ring opening, and decarboxylation. The reactivity of the 5-carboxyl group (5-CO) and 2-carboxyl group (2-CO) in the carbonyl addition step was examined, and it was confirmed that the carbonyl addition occurs at 5-CO. It was the first calculation evidence to support carbonyl addition step as rate-determining step.

**FIGURE 2:**
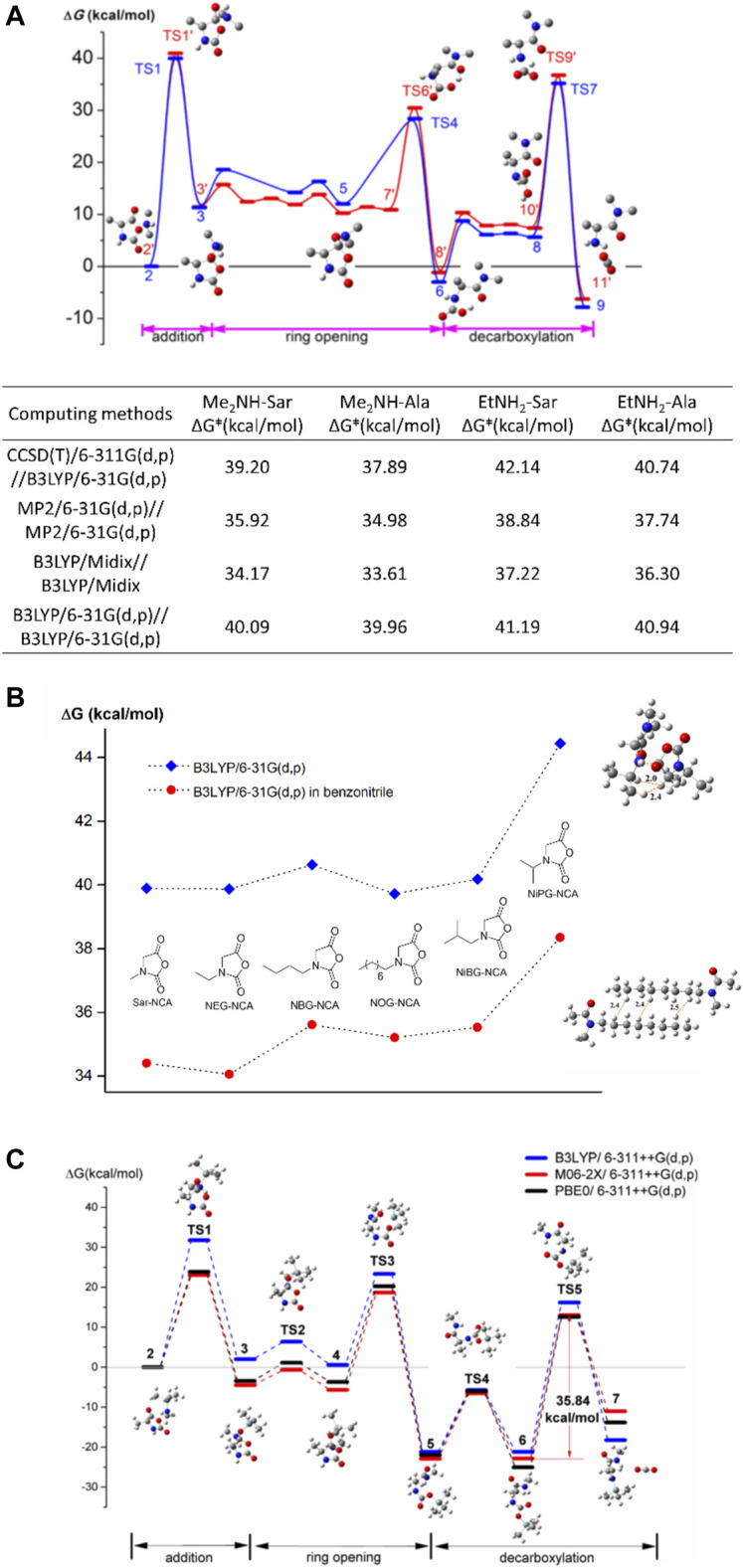
**(A)** The whole profiles of Gibbs free energy barriers in the ring opening polymerization of Ala-NCA initiated by dimethylamine and ethylamine with 3-dimensional models. Some hydrogen atoms are omitted for clarity. Thermodynamics and kinetic data, calculated by four basis set methods of energy barrier are listed. **(B)** The Gibbs free energy barriers in carbonyl addition step of six NNCAs in a vacuum and solution of benzonitrile. Transition states on the carbonyl addition step of NiPG-NCA and optimized geometry of an aggregated dimer with octyl side groups are illustrated with the distance between some hydrogen pairs in the unit of Å. Dash line is used as a guide. **(C)** The whole profiles of Gibbs free energies in ring opening polymerization of Ala-NCA initiated by MeNHTMS in benchmark. Reproduced with permission from corresponding references.

After confirming the rate-determining step and three-step framework in the ROP of Ala-NCA initiated by ethylamine, it is of interest whether the NNCAs will follow the same mechanism. Compared with NCA, propagation end in NNCA ROP is a secondary amine rather than a primary amine due to the substitution on nitrogen, which may influence the whole NAM mechanism in both the carbonyl addition and decarboxylation steps and further change the rate-determining step. The DFT calculations of polymerizations of Ala-NCA as well as sarcosine-NCA (Sar-NCA) (Liu and Ling, 2015) are investigated with two initiators, i.e., primary amine and secondary amine with weak steric hindrance. The rate-determining steps in ROPs of NCA and NNCA initiated by either ethyl amine or dimethylamine, are the amine addition on the 5-carbonyl group rather than decarboxylation as before. These results are also benchmarked by Midix, Møller−Plesset perturbation theory, and coupled cluster theory, as shown in Figure 2A.

A small energy barrier difference was found in the carbonyl addition step between primary and secondary amine initiated cases. Based on the energy barrier, the reactivity ratios (*r*) of Ala-NCA and Sar-NCA are obtained with the product *r*_1_ × *r*_2_ = 1. Considering that ethylamine or dimethylamine are not only initiators but a simplified model of propagating end in ROP. The calculation results indicate that the random polymerization of NNCA and NCA is available especially for Ala-NCA and Sar-NCA. However, although the block copolymer of polypeptides-*co*-polypeptoids was obtained from both polypeptide and polypeptoid ends *via* sequential monomer feeding process (Birke et al., 2014; Heller et al., 2015), the synthesis of random poly(peptide-*r*-peptoid) has not yet been realized in experiments.

In the ROP of NNCA monomers, Sar-NCA has shown good reactivity in both experimental results and DFT calculations. Do NNCAs with large substituted groups have a similar polymerization mechanism to that of Sar-NCA? The side group effect in NNCA is of interest since the substituent group is related to the physical properties of polypeptoids such as hydrophilicity and crystallinity. In an experimental kinetic investigation (Fetsch et al., 2011) the ROP of NNCA follows pseudo first-order kinetics, and the apparent polymerization rate of NNCA is in the order of Sar >> EtGly > PrGly > nBuGly > iBuGly in benzonitrile. In the DFT calculation of the ROP of six NNCA monomers, the propagating chain ends rather than employing simple secondary amines to model real propagation (Bai and Ling, 2019). The rate-determining steps in all NNCA cases are still carbonyl addition in a three-step framework, but the energy barriers in the carbonyl addition step highly depend on the branched structure of substituents, as shown in Figure 2B. The steric hindrance causes strong repulsion of hydrogen pairs in carbonyl addition step, which leads to low reactivity of the *β*-branched amino acid such as NiPG-NNCA. In cases of linear and *γ*-C branched NNCAs, aggregation of side groups is observed in DFT calculations, which is considered responsible for low reaction rate instead of steric hindrance.

### Other Labile Groups: NAM-TMS Mechanism

Can any group be the transfer moiety instead of proton in NAM? Trimethylsilyl (TMS) containing compounds, such as TMS-amines(Lu and Cheng, 2007, 2008; Baumgartner et al., 2017), have been found to successfully initiate living/controlled ROP of NCAs. DFT calculations have been carried out for the mechanism in ROP of Ala-NCA and Sar-NCA initiated by TMS amine with MeNHTMS as a model compound of initiator (Bai and Ling, 2017). In MeNHTMS, both proton and TMS groups can be transfer moiety in NAM. According to our DFT results, TMS transfer is thermodynamically favored compared with proton transfer in a benchmark of three functionals, as shown in Figure 2C. The mechanism is named as the “*NAM-TMS mechanism*”. In NAM-TMS, the CO_2_-TMS bearing compound is noteworthy as it has been observed to have great stability in both calculation and experimental work, which enlarges the energy barrier in the decarboxylation step, which becomes the rate-determining step. NAM-TMS is the first calculation evidence to support decarboxylation as a rate-determining step in NAM.

### α,ω-Telechelic Amines

When α,ω-telechelic functionalized amines are employed as initiators, the end group of obtained PAA can be designed *via* adjusting interaction between two functionalized groups. Compared with NCA, NTA is more tolerable for water, hydroxyl, and thiol groups, which enables the convenient synthesis of α,ω-telechelic functionalized PAAs, such as ROP of NTA initiated α,ω-aminoalcohol(Tao et al., 2017) and cysteamine. (Tao et al., 2018b). In the case of some aminoalcohols, i.e., 2-amino-1-ethanol, 3-amino-1-propanol, and 4-aminomethylbenzyl alcohol, DFT calculations confirm that hydrogen bonding leads to the activation of hydroxyl group *via* 5- or 6-membered ring or π–π stacking in the initiation, and produces polypeptoid mixtures. By contrast, it is hard to activate thiol groups by amino groups, and the α,ω-cysteamine-initiated ROP selectively results in pure thiol-capped polypeptoids.

## Summary and Perspectives

In amine-mediated ROP of NCA and NTA, integrated understanding of NAM has been established by DFT calculations including three-step framework, NAM-TMS mechanism, the effect of *N*-substitution in initiator or monomer ring, and the α,ω-functionalized initiator. Besides NAM, two problems have still not been solved in the ROP of NCA and NTA. The other mechanism, i.e., active monomer mechanism, is still not pieced in the whole picture puzzle. AMM contains more elemental reactions and more complicated kinetics than NAM. Another mechanism problem lies in the effect of chain structure, e.g., the acceleration induced by helix secondary structures (Baumgartner et al., 2017; Song et al., 2019) or macroinitiators.

## References

[B1] Aubert P. Knott E. B. (1950). Synthesis of thiazolid-2:5-dione. Nature 166, 1039–1040. 10.1038/1661039b0 14796688

[B2] Bai T. Ling J. (2017). NAM-TMS mechanism of α-amino acid N-carboxyanhydride polymerization: a DFT study. J. Phys. Chem. A. 121 (23), 4588–4593. 10.1021/acs.jpca.7b04278 28524664

[B3] Bai T. Ling J. (2019). Polymerization rate difference of N‐alkyl glycine NCAs: steric hindrance or not? Biopolymers 14, e23261. 10.1002/bip.23261 30747994

[B4] Bai T. Shen B. Cai D. Luo Y. Zhou P. Xia J. (2020). Understanding ring-closing and racemization to prepare α-amino acid NCA and NTA monomers: a DFT study. Phys. Chem. Chem. Phys. 22 (26), 14868–14874. 10.1039/d0cp01174f 32582885

[B5] Baumgartner R. Fu H. Song Z. Lin Y. Cheng J. (2017). Cooperative polymerization of α-helices induced by macromolecular architecture. Nat. Chem. 9 (7), 614–622. 10.1038/Nchem.2712 28644469

[B6] Birke A. Huesmann D. Kelsch A. Weilbaecher M. Xie J. Bros M. (2014). Polypeptoid-block-polypeptide copolymers: synthesis, characterization, and application of amphiphilic block copolypept(o)ides in drug formulations and miniemulsion techniques. Biomacromolecules 15 (2), 548–557. 10.1021/bm401542z 24354284

[B7] Birke A. Ling J. Barz M. (2018). Polysarcosine-containing copolymers: synthesis, characterization, self-assembly, and applications. Prog. Polym. Sci. 81, 163–208. 10.1016/j.progpolymsci.2018.01.002

[B8] Cao J. B. Siefker D. Chan B. A. Yu T. Y. Lu L. Saputra M. A. (2017). Interfacial ring-opening polymerization of amino-acid-derived N-thiocarboxyanhydrides toward well-defined polypeptides. Acs Macro Lett. 6 (8), 836–840. 10.1021/acsmacrolett.7b00411

[B9] Coleman D. Farthing A. C. (1950). Synthetic polypeptides. Part II. properties of oxazolid-2-5-diones and an initial study of the preparation of polypeptides therefrom. J. Chem. Soc. 23, 3218–3222. 10.1039/jr9500003218

[B10] Deming T. J. (1997). Facile synthesis of block copolypeptides of defined architecture. Nature 390 (6658), 386–389. 10.1038/37084 9389476

[B11] Deming T. J. (2016). Synthesis of side-chain modified polypeptides. Chem. Rev. 116 (3), 786–808. 10.1021/acs.chemrev.5b00292 26147333

[B12] Falivene L. Al Ghamdi M. Cavallo L. (2016). Mechanistic insights into the organopolymerization of N-methyl N-carboxyanhydrides mediated by N-heterocyclic carbenes. Macromolecules 49 (20), 7777–7784. 10.1021/acs.macromol.6b01722

[B13] Falivene L. Cavallo L. (2017). Guidelines to select the N-heterocyclic carbene for the organopolymerization of monomers with a polar group. Macromolecules 50 (4), 1394–1401. 10.1021/acs.macromol.6b02646

[B14] Fan J. Zou J. He X. Zhang F. Zhang S. Raymond J. E. (2014). Tunable mechano-responsive organogels by ring-opening copolymerizations of N-carboxyanhydrides. Chem. Sci. 5 (1), 141–150. 10.1039/c3sc52504j PMC3865608 24363890

[B15] Farthing A. C. Reynolds R. J. W. (1950). Anhydro-N-carboxy-DL-beta-phenylalanine. Nature 165 (4199), 647. 10.1038/165647a0 15416749

[B16] Fetsch C. Grossmann A. Holz L. Nawroth J. F. Luxenhofer R. (2011). Polypeptoids from N-substituted glycine N-carboxyanhydrides: hydrophilic, hydrophobic, and amphiphilic polymers with Poisson distribution. Macromolecules 44 (17), 6746–6758. 10.1021/ma201015y

[B17] Gangloff N. Ulbricht J. Lorson T. Schlaad H. Luxenhofer R. (2016). Peptoids and polypeptoids at the Frontier of supra- and macromolecular engineering. Chem. Rev. 116 (4), 1753–1802. 10.1021/acs.chemrev.5b00201 26699377

[B18] Guo L. Zhang D. (2009). Cyclic poly(α-peptoid)s and their block copolymers from N-heterocyclic carbene-mediated ring-opening polymerizations of N-substituted N-carboxylanhydrides. J. Am. Chem. Soc. 131 (50), 18072–18074. 10.1021/ja907380d 19950948

[B19] Habraken G. J. M. Wilsens K. Koning C. E. Heise A. (2011). Optimization of N-carboxyanhydride (NCA) polymerization by variation of reaction temperature and pressure. Polym. Chem. 2 (6), 1322–1330. 10.1039/c1py00079a

[B20] Heller P. Mohr N. Birke A. Weber B. Reske-Kunz A. Bros M. (2015). Directed interactions of block copolypept(o) ides with mannose-binding receptors: peptomicelles targeted to cells of the innate immune system. Macromol. Biosci. 15 (1), 63–73. 10.1002/mabi.201400417 25560686

[B21] Hirschmann R. Dewey R. Schoenewaldt E. Joshua H. Paleveda W. J. Jr Schwam H. (1971). Synthesis of peptides in aqueous medium. VII. Preparation and use of 2, 5-thiazolidinediones in peptide synthesis. J. Org. Chem. 36 (1), 49–59. 10.1021/jo00800a013 5543046

[B22] Kricheldorf H. R. (1987). α-Amino acid N-carboxyanhydrides and related heterocycles. Berlin, Heildelberg, New York: Springer Pub.

[B23] Kricheldorf H. R. Bösinger K. (1976). Mechanismus der NCA-polymerisation, 3. Über die amin katalysierte polymerisation von sarkosin-NCA und -NTA. Makromol. Chem. 177 (5), 1243–1258. 10.1002/macp.1976.021770502

[B24] Kricheldorf H. R. (2006). Polypeptides and 100 years of chemistry of α‐amino acid N‐carboxyanhydrides. Angew. Chem. Int. Ed. 45 (35), 5752–5784. 10.1002/anie.200600693 16948174

[B25] Leuchs H. Geiger W. (1908). Über die Anhydride von α‐Amino‐N‐carbonsäuren und die von α‐Aminosäuren. Ber. Dtsch. Chem. Ges. 41 (2), 1721–1726. 10.1002/cber.19080410232

[B26] Leuchs H. (1906). Glycine-carbonic acid. Ber. Dtsch. Chem. Ges. 39, 857–861. 10.1002/cber.190603901133

[B27] Leuchs H. Manasse W. (1907). Über die Isomerie der Carbäthoxyl‐glycyl glycinester. Ber. Dtsch. Chem. Ges. 40 (3), 3235–3249. 10.1002/cber.19070400387

[B28] Ling J. Huang Y. (2010). Understanding the ring-opening reaction of α-amino acid N-carboxyanhydride in an amine-mediated living polymerization: a DFT study. Macromol. Chem. Phys. 211 (15), 1708–1711. 10.1002/macp.201000115

[B29] Ling J. Peng H. Shen Z. (2012). Deprotonation reaction of α-amino acid N-carboxyanhydride at 4-CH position by yttrium tris[bis(trimethylsilyl)amide]. J. Polym. Sci. Part. A: Polym. Chem. 50 (18), 3743–3749. 10.1002/pola.26156

[B30] Liu J. Ling J. (2015). DFT Study on amine-mediated ring-opening mechanism of α-amino acid N-carboxyanhydride and N-substituted glycine N-carboxyanhydride: secondary amine versus primary amine. J. Phys. Chem. A. 119 (27), 7070–7074. 10.1021/acs.jpca.5b04654 26086174

[B31] Lu H. Cheng J. (2007). Hexamethyldisilazane-mediated controlled polymerization of alpha-Amino acid N-carboxyanhydrides. J. Am. Chem. Soc. 129 (46), 14114–14115. 10.1021/ja074961q 17963385

[B32] Lu H. Cheng J. (2008). N-trimethylsilyl amines for controlled ring-opening polymerization of amino acid N-carboxyanhydrides and facile end group functionalization of polypeptides. J. Am. Chem. Soc. 130 (38), 12562–12563. 10.1021/ja803304x 18763770

[B33] Mazo A. R. Allison-Logan S. Karimi F. Chan N. J.-A. Qiu W. Duan W. (2020). Ring opening polymerization of α-amino acids: advances in synthesis, architecture and applications of polypeptides and their hybrids. Chem. Soc. Rev. 49 (14), 4737–4834. 10.1039/C9CS00738E 32573586

[B34] Montalbetti C. A. Falque V. (2005). Amide bond formation and peptide coupling. Tetrahedron 61 (46), 10827–10852. 10.1016/j.tet.2005.08.031

[B35] Peng H. Ling J. Shen Z. (2012). Ring opening polymerization of α-amino acid N-carboxyanhydrides catalyzed by rare earth catalysts: polymerization characteristics and mechanism. J. Polym. Sci. Part. A: Polym. Chem. 50 (6), 1076–1085. 10.1002/pola.25848

[B36] Shalitin Y. Katchalski E. (1960). Amine initiated copolymerization of N-Carboxy-α-amino acid Anhydrides1. J. Am. Chem. Soc. 82 (7), 1630–1636.

[B37] Siefker D. Williams A. Z. Stanley G. G. Zhang D. (2018). Organic acid promoted controlled ring-opening polymerization of α-amino acid-derived N-thiocarboxyanhydrides (NTAs) toward well-defined polypeptides. ACS Macro Lett. 7 (10), 1272–1277. 10.1021/acsmacrolett.8b00743

[B38] Song Z. Fu H. Wang J. Hui J. Xue T. Pacheco L. A. (2019). Synthesis of polypeptides via bioinspired polymerization of *in situ* purified N-carboxyanhydrides. Proc. Natl. Acad. Sci. USA 116 (22), 10658–10663. 10.1073/pnas.1901442116 31088971 PMC6561217

[B39] Szwarc M. (1965). The kinetics and mechanism of N-carboxy-α-amino-acid anhydride (NCA) polymerisation to poly-amino acids. Fortschritte der Hochpolymeren-forschung. Berlin: Springer, 1–65.

[B40] Tao X. Deng Y. Shen Z. Ling J. (2014). Controlled polymerization of N-substituted glycine N-thiocarboxyanhydrides initiated by rare earth borohydrides toward hydrophilic and hydrophobic polypeptoids. Macromolecules 47 (18), 6173–6180. 10.1021/ma501131t

[B41] Tao X. Li M.-H. Ling J. (2018a). α-Amino acid N-thiocarboxyanhydrides: a novel synthetic approach toward poly(α-amino acid)s. Eur. Polym. J. 109, 26–42. 10.1016/j.eurpolymj.2018.08.039

[B42] Tao X. Zheng B. Bai T. Li M.-H. Ling J. (2018b). Polymerization of N-substituted glycine N-thiocarboxyanhydride through regioselective initiation of cysteamine: a direct way toward thiol-capped polypeptoids. Macromolecules 51 (12), 4494–4501. 10.1021/acs.macromol.8b00259

[B43] Tao X. Zheng B. Bai T. Zhu B. Ling J. (2017). Hydroxyl group tolerated polymerization of N-substituted glycine N-thiocarboxyanhydride mediated by aminoalcohols: a simple way to α-hydroxyl-ω-aminotelechelic polypeptoids. Macromolecules 50 (8), 3066–3077. 10.1021/acs.macromol.7b00309

[B44] Vinick F. J. Jung S. (1982). Concerning the preparation of optically pure N-(thiocarboxy)-L-aspartic anhydride. J. Org. Chem. 47 (11), 2199–2201. 10.1021/jo00132a046

[B45] Wu Y. Zhang D. Ma P. Zhou R. Hua L. Liu R. (2018). Lithium hexamethyldisilazide initiated superfast ring opening polymerization of alpha-amino acid N-carboxyanhydrides. Nat. Commun. 9 (1), 5297. 10.1038/s41467-018-07711-y 30546065 PMC6294000

[B46] Yuan J. Sun Y. Wang J. Lu H. (2016). Phenyl trimethylsilyl sulfide-mediated controlled ring-opening polymerization of α-amino acid N-carboxyanhydrides. Biomacromolecules 17 (3), 891–896. 10.1021/acs.biomac.5b01588 26796118

[B47] Yuan J. Zhang Y. Li Z. Wang Y. Lu H. (2018). A S-Sn Lewis pair-mediated ring-opening polymerization of α-amino acid N-carboxyanhydrides: fast kinetics, high molecular weight, and facile bioconjugation. ACS Macro Lett. 7 (8), 892–897. 10.1021/acsmacrolett.8b00465 35650961

[B48] Zhao W. Lv Y. Li J. Feng Z. Ni Y. Hadjichristidis N. (2020). A synthetic methodology of site‐specific functionalized polypeptides: metal‐free, highly active and selective at room temperature. Angew. Chem. Int. Ed. 11 (59), 2–9. 10.1002/anie.202009316 32935922

[B49] Zhao W. Lv Y. Li J. Feng Z. Ni Y. Hadjichristidis N. (2019). Fast and selective organocatalytic ring-opening polymerization by fluorinated alcohol without a cocatalyst. Nat. Commun. 10 (1), 3590. 10.1038/s41467-019-11524-y 31399569 PMC6689068

[B50] Zou J. Fan J. He X. Zhang S. Wang H. Wooley K. L. (2013). A facile glovebox-free strategy to significantly accelerate the syntheses of well-defined polypeptides by N-carboxyanhydride (NCA) ring-opening polymerizations. Macromolecules 46 (10), 4223–4226. 10.1021/ma4007939 23794753 PMC3686519

